# Colostrum-Induced Temporary Changes in the Expression of Proteins Regulating the Epithelial Barrier Function in the Intestine

**DOI:** 10.3390/foods11050685

**Published:** 2022-02-25

**Authors:** Sylwia Rzeszotek, Grzegorz Trybek, Maciej Tarnowski, Karol Serwin, Aleksandra Jaroń, Gabriela Schneider, Agnieszka Kolasa, Barbara Wiszniewska

**Affiliations:** 1 Department of Histology and Embryology, Pomeranian Medical University in Szczecin, 72 Powstańców Wlkp., 70-111 Szczecin, Poland; agnieszka.kolasa@pum.edu.pl (A.K.); barbara.wiszniewska@pum.edu.pl (B.W.); 2 Department of Oral Surgery, Pomeranian Medical University in Szczecin, 72 Powstańców Wlkp., 70-111 Szczecin, Poland; g.trybek@gmail.com (G.T.); aleksandra.jaron@pum.edu.pl (A.J.); 3 Department of Physiology, Pomeranian Medical University in Szczecin, 72 Powstańców Wlkp., 70-111 Szczecin, Poland; maciej.tarnowski@pum.edu.pl; 4 Department of Infectious Tropical Diseases and Immune Deficiency, Pomeranian Medical University in Szczecin, Arkońska 4, 71-455 Szczecin, Poland; karolserwin@gmail.com; 5 UofL Health-Brown Cancer Center and Division of Medical Oncology and Hematology, Department of Medicine, University of Louisville, Louisville, KY 40202, USA; gabriela.schneider@louisville.edu

**Keywords:** colostrum, lactoferrin, intestine, cellular junctions

## Abstract

The intestinal wall and epithelial cells are interconnected by numerous intercellular junctions. Colostrum (Col), in its natural form, is a secretion of the mammary gland of mammals at the end of pregnancy and up to 72 h after birth. Recently, it has been used as a biologically active dietary supplement with a high content of lactoferrin (Lf). Lf, a glycoprotein with a broad spectrum of activity, is becoming more popular in health-promoting supplements. This study aims to investigate whether Col supplementation can affect small and large intestine morphology by modulating the expression of selected proteins involved in tissue integrity. We examined the thickness of the epithelium, and the length of the microvilli, and assessed the expression of *CDH1*, *CDH2*, *CTNNB*, *CX43*, *VCL*, *OCLN*, *HP*, *MYH9*, and *ACTG2* gene levels using qRT-PCR and at the protein level using IHC. Additionally, to evaluate whether the effect of Col supplementation is temporary or persistent, we performed all analyses on tissues collected from animals receiving Col for 1, 3, or 6 months. We noticed a decrease in *CDH1* and *CDH2* expression, especially after 3 months of supplementation in the large intestine and in *CTNNB* in the small intestine as well as increased levels of *CX43* and *CTNNB1* in the small intestine. The present data indicate that Col can temporarily alter some components of the cell adhesion molecules involved in the formation of the cellular barrier.

## 1. Introduction

The intestinal barrier is a dynamic structure composed of many elements—epithelial cells, lamina propria, immune cells, gut microbiota, the mucous layer, and, cooperating with them, cell adhesion molecules participating in the formation of cellular junctions (CJ). Data derived from basic and clinical research indicate that structural and functional disturbances of CJ components are involved in the development of several diseases, including those related to the gastrointestinal system, such as inflammatory bowel disease, Crohn’s disease, or cancer [1]. It should be emphasized that cell adhesion molecules are expressed not only on the surface of enterocytes but also on the other cells that make up the intestinal wall, e.g., goblet cells, neuroendocrine cells, Paneth cells, fibroblasts, smooth muscle cells, etc. Many factors can change the expression of intercellular junction proteins, including several supplements such as vitamins A, C, D, and zinc, or pickled food [2].

Recently, Colostrum (Col), a secretion of the mammary gland, is becoming more common in health-promoting supplements. Colostrum bovinum is characterized by a high lactoferrin (Lf) concentration (an iron-binding glycoprotein with a broad spectrum of activity), and, among others, immune factors (immunoglobulins), vitamins and minerals, growth factors, proline-rich proteins (PRP), lysozyme, carbohydrates, fats, and much more. Lf was shown to have anti-cancer, anti-viral, anti-bacterial, anti-inflammatory [3], and anti-fungal properties [4]. It also ameliorates, *inter alia,* hypertension [5], gastrointestinal ulcers [6], cardiovascular diseases [7,8] or bone regeneration [9]. More importantly, Lf was shown to be an important regulator of the gastrointestinal immune response [10] and can affect the expression of some tight junction (TJ) proteins in vitro [11]. 

Based on the facts above, we hypothesize that bovine Col supplementation can change the expression level of selected genes and proteins of intercellular connections and motor proteins of the small and large intestine in vivo. To address this question, we evaluated the morphological changes of the small and large intestines of Col-supplemented animals. Moreover, we analyzed the expression patterns of some proteins of occludens, adherens, and nexus intercellular junctions: cadherin-1 (epithelial cadherin, Cdh1), cadherin-2 (neural cadherin, Cdh2), connexin 43 (Cx43), vinculin (Vcl), catenin beta-1 (Ctnnb1), haptoglobin (Hp), occludin (Ocln), and selected motor proteins, such as non-muscle myosin heavy chain 9 (Myh9) and actin gamma smooth muscle 2 (Actg2), both at the protein and mRNA levels. Additionally, we investigate possible correlations in gene expression caused by possible interconnections between them.

## 2. Materials and Methods

### 2.1. Animals and Procedures

**Figure 1:**
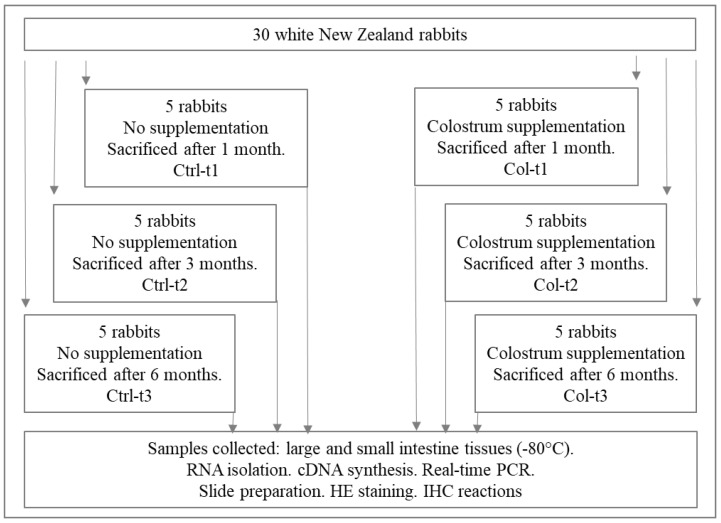
Graphical overview of the procedure for obtaining biological material and division into test and control groups.

The study was conducted in accordance with Polish law and with the approval of the Local Ethics Committee for Scientific Experiments on Animals in Szczecin, Poland (Resolution No. 27/2015, 12 June 2015). Thirty healthy 6-month-old white New Zealand male rabbits with an average weight of 3.5 ± 0.3 kg were recruited in this randomized, blind experimental study. One month before the surgical intervention, all animals were accommodated in separate cages with free access to food and water. Before the start of the experiment, the animals were randomly divided into six groups (three control groups and three experimental groups), with five animals per group. The control groups consisted of the animals not treated with Colostrum Colostrigen from Genactiv (Poznań, Poland) (*n* = 15; Ctrl), while the animals from the supplemented groups (*n* = 15; Lf) received per os an aqueous solution of Colostrum Colostrigen from Genactiv (Poznań, Poland, composition: lyophilized colostrum bovinum) at a dose of 100 mg/kg body weight/day for 1 month (Col-t1), 3 months (Col-t2), and 6 months (Col-t3). Animals were sacrificed after 1 month (Ctrl-t1 and Col-t1), 3 months (Ctrl-t2 and Col-t2), and 6 months (Ctrl-t3 and Col-t3) (Figure 1). The Colostrum dose was chosen based on the published experiences of other researchers, emphasizing that the effects observed were highly dose-dependent. Due to the physiological differences between females and males and the small size of the experimental groups, we decided to conduct analyses in groups composed of males. Thus, we avoided an additional variable in the form of differences of the endocrine system.

### 2.2. Tissues

Small and large intestine samples from each rabbit were collected and kept at −80 °C or fixed in 10% buffered formalin for at least 24 h, but no more than 36 h.

### 2.3. Quantitative Real-Time Reverse Transcription PCR (qRT-PCR) Analysis

Quantitative analyses of mRNA expression of *CDH1*, *CDH2*, *CTNNB1*, *VCL*, *OCLN*, *CX43*, *HP*, *MYH9*, and *ACTG2* were performed in a two-step reverse transcription PCR as described previously [12]. Each sample was analyzed with two technical replicates, and mean Ct values were used for further analysis. The relative quantity of a target, normalized to the endogenous control GAPDH gene and relative to a calibrator, is expressed as 2–ΔCt (-fold difference), where Ct is the threshold cycle, ΔCt = (Ct of target genes) − (Ct of endogenous control gene). The following primer pairs, designed with Primer-Blast were used: CDH1 (5′-TGC ACA GGC CGG AAA CCA G-3′ and 5′-ACG GCC TTC AGC GTG ACC TT-3′), CDH2 (5′-CCG TGG CAG CTG GACT GGA T-3′ and 5′-GATGACGGCCGTGGCTGTGT-3′), CTNNB1 (5′-ATG GAG CCA GAC AGA AAA GC-3′ and 5′-TGG GAG GTA TCC ACA TCC TCT T-3′), VCL (5′-TCT CGC ACC TGG TGA TCA TGC-3′ and 5′-TGA ACA GTC TCT TTT CCA ACC C-3′), OCLN (5′-CGG CGT CGG CAG ATT GA-3′ and 5′-GTG CAT CTC ACC ACC GTA CA-3′), CX43 (5′-ACA AAG TCC AAG CCT ACT CCA-3′ and 5′-CCA GGT TGC TGA GTG TTA CAA-3′), HP (5′-CAT CGG TGG ATC TCT GGA CG-3′ and 5′-GAC AAG ATT GTG GCG GGA GA-3′), MYH9 (5′-GGC CCT GCT GGA TGA GGA AT-3′ and 5′-CCA GCG TAG TGG ATG ATG CAG A-3′), ACTG2 (5′-GCA TGG AGT CTG CTG GGA TT-3′ and 5′-GAG AGG ACGT TGT TGGC GTA-3′), oGAPDH (5′-TGC CAC CCA CTC CTC TAC CTT CG-3′ and oGAPDH 5′-CCG GTG GTT TGA GGG CTC TTA CT-3′). Primers were designed so that the amplification efficiencies ranged from 90% to 110%. The primer sequences used in the study were obtained according to the sequence information obtained from the NCBI database, and were synthesized by Oligo.pl (IBB PAN, Warsaw, Poland).

### 2.4. Histological and Immunohistological Methods

The dissected intestinal tissues were fixed with 10% formalin for at least 24 h, but for no more than 36 h, and then treated as described previously [12]. The slides were incubated for 1 h at room temperature (RT), with primary antibodies against Cdh1 (anti-E-cadherin antibody (G-10), mouse monoclonal, cat# sc-8426, Santa Cruz Biotechnology, Dallas, TX, USA), Cdh2 (anti-*N*-cadherin antibody (D-4), mouse monoclonal, cat# sc-8424, Santa Cruz Biotechnology, Dallas, TX, USA), Ctnnb1 (anti-β-catenin antibody (E-5), cat# sc-7963, mouse monoclonal, Santa Cruz Biotechnology, Dallas, TX, USA), Vcl (anti-vinculin antibody (7F9), cat# sc-73614, mouse monoclonal, Santa Cruz Biotechnology, Dallas, TX, USA), Ocln (anti-occludin antibody (E-5), cat# sc-133256, Santa Cruz Biotechnology, Dallas, TX, USA), and Cx43 (anti-connexin 43 antibody (F-7), cat# sc-271837, Santa Cruz Biotechnology, Dallas, TX, USA). Antibodies were diluted in Antibody Diluent (cat# ab64211, abcam, Cambridge, UK). After washing, slides were covered with ready-to-use EnVision FLEX LINKER for 30 min, RT. To visualize the antigen-antibody complex, the reaction of avidin-biotin-horseradish peroxidase with DAB as a chromogen, according to the included staining procedure instructions, was performed. Sections were washed in distilled H_2_O and counterstained with hematoxylin. For negative control, specimens were processed in the absence of a primary antibody. Positive staining was determined microscopically (Leica DM5000B, Wetzlar, Germany) by visual identification of brown pigmentation.

### 2.5. Statistic Analysis

GraphPad Prism 9 software was used for conducting statistical tests (GraphPad, La Jolla, San Diego, CA, USA). Because of low group counts, we assumed the normal distribution of analyzed genes based on previous observations [13], whereas for epithelium and microvilli measurements, we evaluated QQ plots to confirm that there are no significant deviations from the normal distribution. Two-group contrasts were tested for statistical significance by *t*-test with Welch correction. Differences between more than two groups were tested for statistical significance by one-way Welch’s ANOVA test, followed by the Dunnett T3 comparisons test. Data were visualized using boxplots with whiskers indicating 5th and 95th percentiles. For correlation analysis, all data obtained for the particular parameter from control and tested groups were combined, and normality of distribution was tested using the D’Agostino-Pearson omnibus K2 test at a 5% significance level. Correlation between parameters was evaluated by calculating Pearson (when both analyzed parameters passed normality test) or Spearman (when at least one of the parameters did not pass the normality test) correlation coefficients. Because of the preliminary character of our work, *p*-values were not adjusted for multiple comparisons and should be considered exploratory in nature.

## 3. Results

### 3.1. Small Intestine

#### 3.1.1. Effect of Col Supplementation on the Morphology of the Small Intestine

**Figure 2:**
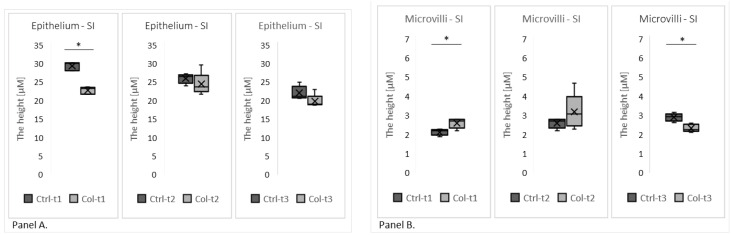
The graphs show the height of the epithelium (**Panel A**) and the microvilli (**Panel B**) in the small intestines of rabbits supplemented with Col for 1, 3, or 6 months and corresponding controls. SI—small intestine. * *p* (two-tailed) ≤ 0.05.

Two group analyses, performed separately at three time points (1 month, 3 months, and 6 months), revealed the Col-induced changes in the morphology of the small intestine epithelium (Figure 2). One month of Col supplementation reduced the height of the epithelium (Col-t1 vs. Ctrl-t1; Figure 2, panel A), but this change was not persistent since it was not found in animals that received Col for 3 or 6 months. Additionally, we observed the elongation of the microvilli in animals receiving Col for 1 month, and, surprisingly, the opposite effect in animals receiving Col supplementation for 6 months (Figure 2, panel B). 

#### 3.1.2. Effects of Col Supplementation on the Expression of Genes Forming the Epithelial Barrier of the Small Intestine

**Figure 3:**
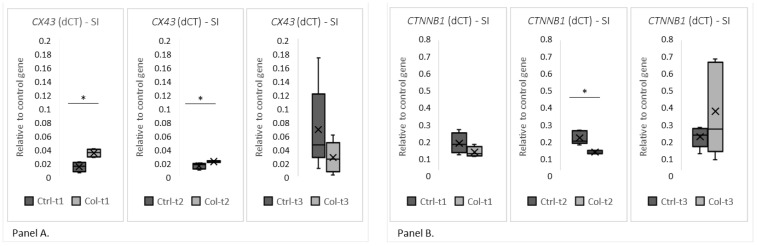
The charts show the deltaCt (dCt), the fold change of gene expression (relative to the housekeeping gene GAPDH) in the small intestine. The (**Panel A**) shows *CX43* and the (**Panel B**) shows *CTNNB*1 expression at all three time points of Col supplementation. SI—small intestine. * *p* (two-tailed) ≤ 0.05.

Two group analyses were performed separately for all time points to evaluate gene expression. A statistically significant increase in the level of *CX43* expression was found in the small intestines of animals receiving Col for 1 and 3 months (Figure 3, panel A), but was not observed after 6 months. Additionally, we have not found any trends in expression patterns of *CX43* in both the Col-treated and control groups. Significant changes in the expression level were also identified in *CTNNB*1 (Figure 3, panel B), where lower expression was detected in animals after 3 months of supplementation with Col. Interestingly, neither short nor long supplementation with Col affected expression patterns of the remaining analyzed genes (*CDH1*, *OCLN*, *HP*, *MYH9*, *VCL*, *ACTG2*, and *CDH2*; Figure S1—supplementary data). 

#### 3.1.3. Effects of Col Supplementation on the Expression of Proteins Forming the Epithelial Barrier of the Small Intestine

**Figure 4:**
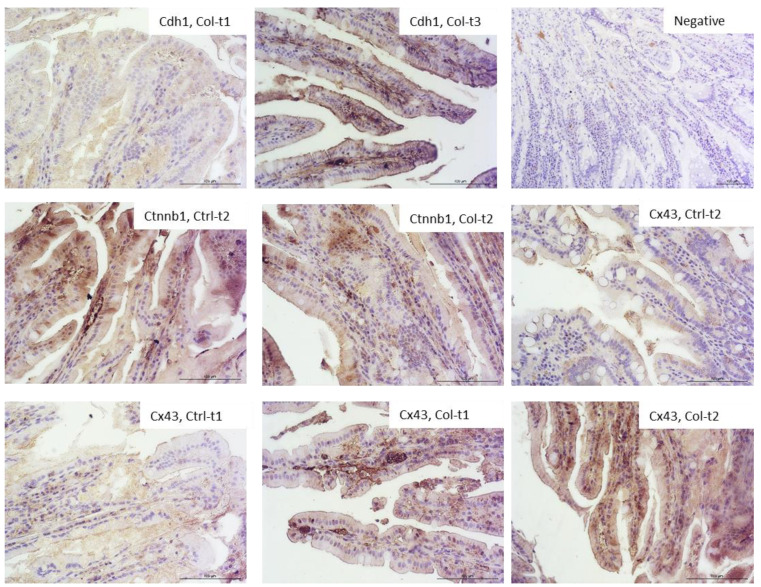
Representative pictures of the protein expression of intercellular junctions in the small intestine derived from control or Col-supplemented animals. We have only selected those where the differences were most apparent (objective magn. ×20, Leica DM5000B, Wetzlar, Germany).

Figure 4 shows the most visible changes in protein expression of the epithelial barrier in the small intestine and negative control. In general, the protein expression of cell adhesion molecules corresponded with the quantitative PCR (qPCR) results. There were no apparent differences in Cdh1, Ocln, Hp, Myh9, Vcl, Actg2, and Cdh2 and no differences after 6 months of treatment in all groups. In the small intestine, the most apparent differences were observed in the level of Cx43 protein after 1 and 3 months of supplementation. Similarly, we observed stronger staining for the Ctnnb protein—however, to a lesser degree than in the case of Cx43. Interestingly, the differences in Cdh1 levels were only observed in the Ctrl group, between Ctrl-t1 and Ctrl-t3.

#### 3.1.4. Correlations between Gene Expression and Small Intestine Epithelium and Microvilli Morphology

A correlation analysis in the small intestine showed a relationship between the expression of the *ACTG2* and *CX43* genes and the height of the epithelium. However, the correlations are moderate, negative for *ACTG2* and positive for *CX43*. The expression of *MYH9* positively correlates with the evaluated genes—moderately with *CDH1*, *CDH2*, *VCL*, *HP*, and *OCLN*, and strongly with *CTNNB*1 (0.72), *ACTG2* (0.76), and *CX43* (0.77). A moderate negative correlation was noticed between *MYH9* and the height of the epithelium. *OCLN* only showed a moderate negative correlation with *CTNNB*1. The expression of *HP* was moderately positively correlated with *CDH2*, *CTNNB*1, and *VCL*, and a stronger positive correlation was observed with *CX43* (0.83) and *ACTG2* (0.81). *VCL* is only correlated in a moderate way with *CDH2*, *CTNNB*1, and *ACTG2*. *ACTG2* moderately correlates with *CDH2* (0.74) and *CTNNB*1. *CTNNB*1 and *CDH1* are also moderately correlated (Table 1).

### 3.2. Large Intestine

#### 3.2.1. Effect of Col Supplementation on the Morphology of the Large Intestine

**Figure 5:**
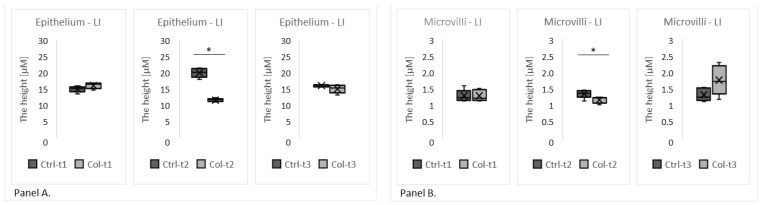
The charts show the height of the epithelium (**Panel A**) and microvilli (**Panel B**) in the large intestine of rabbits treated with Col for 1, 3, or 6 months matched with the appropriate time point control. * *p* (two-tailed) ≤ 0.05.

Two group analyses, performed separately for each time point, revealed Col-induced changes in the morphology of the large intestine epithelium (Figure 5). However, only the group with 3 months of Col supplementation had a reduced height of the epithelium (Col-t2 vs. Ctrl-t2; Figure 5, panel A), but this change was not persistent since it was not found in animals that received Col for 6 months.

#### 3.2.2. Effects of Col Supplementation on the Expression of Genes Forming the Epithelial Barrier of the Large Intestine

**Figure 6:**
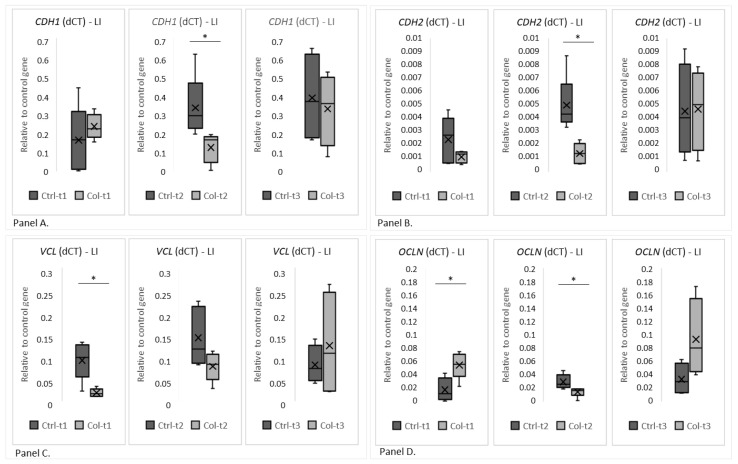
The charts show the dCt, the fold change of gene expression (relative to the housekeeping gene GAPDH) in the large intestine. (**Panel A**) shows *CDH1*, (**Panel B**) shows *CDH2*, (**Panel C**) *VCL* and (**Panel D**) *OCLN*. LI—large intestine. * *p* (two-tailed) ≤ 0.05.

Two group analyses were performed separately for all time points to evaluate gene expression. A statistically significant decrease in the expression of *CDH1* between the Ctrl and Col-supplemented groups was visible after only 3 months (Figure 6, panel A, Ctrl-t2 vs. Col-t2). Also, after 3 months of Col administration, the expression of *CDH2* was significantly lower in the large intestine (Figure 6, panel B, Ctrl-t2 vs. Col-t2) when compared with control. A statistically significant decrease in the level of *VCL* after the administration of Col was only noted between the treated and control groups after 1 month of supplementation (Figure 6, panel C, Ctrl-t1 vs. Col-t1). However, a similar trend was also observed after 3 months of treatment (Figure 5, panel C, Ctrl-t2 vs. Col-t2), but it wasn’t statistically significant. Statistically significant changes in the level of *OCLN* were recorded after 1 and 3 months of administration (Figure 6, panel D, Ctrl-t1 vs. Col-t1 and Ctrl-t2 vs. Col-t2). Interestingly, 1 month of Col treatment increased *OCLN* expression (Col-t1) compared to control (Ctrl-t1), while after 3 months there was a significant decrease in expression compared to control (Ctrl-t2). There was also a significant decrease in the level of *OCLN* expression between the Col supplemented groups after 1 and 3 months (Col-t1 vs. Col-t2). Interestingly, neither short nor long supplementation with Col affected the expression patterns of the remaining analyzed genes (*CTNNB*1, *ACTG2*, *CX43* and *HP*; Figure S2—supplementary data).

#### 3.2.3. Effects of Col Supplementation on the Expression of Proteins Forming the Epithelial Barrier of the Large Intestine

**Figure 7:**
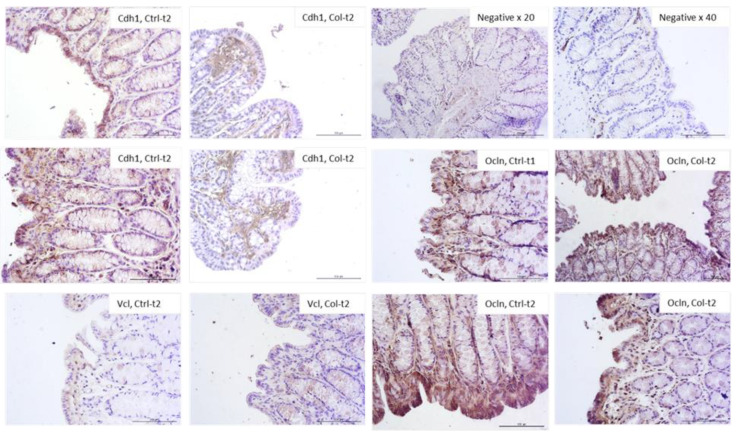
Representative pictures of protein expression of intercellular junctions. Only those where the differences were most apparent were selected (objective magn. ×20, Leica DM5000B, Wetzlar, Germany).

Figure 7 shows the most visible changes in the protein expression pattern of the epithelial barrier in the large intestine of Col-supplemented and control animals. In general, the protein expression of cell adhesion molecules corresponded with the qPCR results. The most apparent differences were observed in Ocln expression after 1 month (increased) and 3 months (decreased) of Col treatment. In both Cdh1 and Cdh2, the protein expression was lower in Col-treated groups compared to the control; however, this change was not persistent after only 3 months of treatment. In Vcl there was also a difference, although not so pronounced. There were no significant differences in the expression of Ctnnb, Actg2, Hp, and Cx43 after 3 or 6 months of supplementation in all groups.

#### 3.2.4. Correlations between Gene Expression and Epithelium or Microvilli in the Large Intestine

A correlation analysis in the large intestine showed a relationship between the expression of the *CDH1*, *CDH2* and *OCLN* genes and the height of the epithelium. However, the strongest positive correlation exists between *CDH1* and *OCLN*. The *CDH2* calculations showed the strongest positive correlation with *VCL*; the remaining weaker relationships were found between *CDH2* and *ACTG2*, *CX43* and epithelial height. For *CTNNB*1, a medium positive correlation exists with *CDH1* and *VCL*. *CX43* forms a positive correlation with *HP*. Our analyses showed an interesting correlation of *OCLN* with the height of the epithelium and the length of the microvilli (Table 2).

## 4. Discussion

### 4.1. Effects of Col Supplementation on Cellular Junction Genes and Protein Expression 

#### 4.1.1. Cdh1 and Cdh2

Cdh1 was found to maintain the intestinal cells’ mechanical integrity and control of the maturation of Paneth and goblet cells [14]. Altered expression of the *CDH1* gene is known as a factor of epithelial to mesenchymal transition (EMT), where reduction of *CDH1* is associated with increased motility and invasiveness of cancer cells and, hence, a worse prognosis. Additionally, there are many connections between Cdh1 and the Wnt/β-catenin signaling pathway [15]. The results of our study showed that Col supplementation did not affect the expression of *CDH1* in the small intestine; however, in the large intestine, after 3 months of treatment with Col (Col-t2), a significantly lower expression was observed compared to the control (Ctrl-t2). This result is surprising, as some cell lines (e.g., the prostate cancer cell line) stimulated with Lf show increased *CDH1* expression and it was demonstrated that this correlates with a better prognosis [16].

In the rat model of induced inflammatory bowel disease (IBD), expression of *CDH1* and Cdh1 was decreased. In vitro analysis showed that miR-155, a major post-transcriptional player in gene expression associated with IBD, suppresses the Cdh1 protein and increases monolayer permeability [17]. Decreased Cdh1 protein expression additionally facilitates neutrophil transmigration, and hence induces a proinflammatory environment. On the other hand, adhesive interactions between the epithelial cells and intraepithelial lymphocytes can be mediated by, inter alia, Cdh1 [18]. Based on the literature, the possible mechanism of temporarily decreased expression of *CDH1* could be related to an Lf-dependent increase in the matrix metalloproteinase 2 (MMP-2) level, which was found to be involved in Cdh1 cleavage (creates soluble Cdh1, sE-cad) [19,20]. Cdh1 should not be discussed in isolation from Cdh2 because of the so-called cadherin switch.

A previous study demonstrated that Cdh2 can substitute Cdh1 during intestinal development; however, this replacement can lead to some disorders, e.g., polyp formations [21]. Moreover, expression of Cdh1 without Cdh2 co-expression can induce neoplastic transformations [21]. Cooperation between Cdh1 and Cdh2 proteins is crucial for proper tissue function [22] and our data showed a moderate positive relationship between *CDH1* and *CDH2* gene expression. While the decreased Cdh1 level during Col supplementation might be worrying, it should be noted that it is not accompanied by an increase in Cdh2 expansion. On the contrary, we noticed a significant decline in *CDH2* gene expression. In our studies, we also found a statistically significant lower expression of *CDH2* after 3 months of Col administration (Col-t2) in the large intestine. Some in vitro studies (based on ATDC5 chondroprogenitor cells) showed that in some chondrogenic cell lines Lf can impair Cdh2 protein expression, as Lf is known to be a bone growth factor [23].

#### 4.1.2. Ctnnb1

Surprisingly, we found no differences in *CTNNB* expression, despite published data indicating that Lf administration can exhibit protective effects against epithelial damage by reducing inflammation and stimulating cell proliferation and, thereby, the Wnt/*CTNNB* pathway [24]. Ctnnb is also associated with a prolonged duration of symptoms in irritable bowel syndrome (IBS) [25]. However, the results of our study indicate a lack of association between *CTNNB* and *CDH2* expression [23]. Therefore, we can conclude that despite some temporary changes in *CDH1* and *CDH2* expression, the *CTNNB* level, as a component of the canonical pathway, was not affected by Col supplementation.

#### 4.1.3. Cx43

Pro-inflammatory mediators can change the expression of connexins and their localization within the junctional complex and Lf is known for its anti-inflammatory properties. Interestingly, down regulation of protein and/or mRNA of Cx43/*CX43*, as well as its increased cytoplasmic localization, was observed in many gastric diseases (for example, *Helicobacter pylori* infections or IBS) [26]. This is interesting as we observed an increased expression of *CX43* after Col treatment in the small intestine after 1 and 3 months of supplementation (Col-t1 and Col-t2). In general, it is thought that inflammatory conditions predispose to the loss of junctional complexes. Cx43 can also change its localization. This transmigration probably facilitates the communication between the intestinal epithelial cells and infiltrating immune cells [27]. In our experiment, we found a strong positive linear relationship between *CDH2* and *CX43* in the small intestine, while in the large intestine, this correlation was moderately positive. One of the mechanisms connecting *CDH2* and *CX43* is that *CX43* uses its isoform to control transcriptional regulation of *CDH2* by direct interaction with transcription factors (and polymerase III) [28].

#### 4.1.4. Vcl

Vcl is an actin-binding protein involved in cell–cell and cell–matrix junctions. Additionally, Vcl is a direct target for some bacteria during cell invasion [29]. Experiments on human osteoblast-like Saos-2 cells showed that on the surfaces coated by collagen and Lf, cells could better develop Vcl-containing focal adhesion plaques [30]. Cited by Adler and McMahan [31], and proposed by Pimental et al. [32], the mechanism of IBS draws attention to cytolethal distending toxins produced by infecting bacteria, which result in the production of anti-toxin antibodies. Due to similarities between toxins and Vcl, antibodies will target not only toxins but also Vcl expressed on interstitial cells of Cajal and myenteric ganglia. Those processes result in gastrointestinal dysmotility and small intestinal bacterial overgrowth. It is not clear whether anti-Vcl autoantibodies are pathogenic or whether they are just a marker of gastrointestinal dysfunction. In our study, we found no differences in Vcl (genes and proteins) expression in the small intestine; however, in the large intestine, we observed decreased expression after 1 month of Col supplementation, but no changes after 3 and 6 months of treatment (Col-t2, Col-t3). Vcl studies carried out by different teams revealed some discrepancies. For example, in some patients with gastric cancers, the expression of Vcl was related to a better prognosis [33]. On the other hand, Vcl was found to promote the proliferation and migration of gastric cancer and serves as a predictor of a poor prognosis in patients [34]. The functional role of Vcl is still unclear, but it was demonstrated that in the absence of Vcl, the expression of Cdh1 on the cell surface is decreased, while the total Cdh1 levels remained unchanged. It was concluded that Vcl can regulate Cdh1 interactions via Ctnnb.

#### 4.1.5. Ocln

The expressions of Ocln (gene and protein) were found to depend on the circadian rhythm in the large intestine [35]. The samples in our study were collected at the same time of day for this reason. It was also demonstrated that stressed animals can show lower expressions of Ocln and *OCLN*. An experiment conducted by Hu et al. [36] showed that treatment with Lf in early life can increase the occurrence of beneficial bacteria and simultaneously decrease the occurrence of potentially pathogenic bacteria in piglets. Bacteria produce short-chain fatty acids (SCFA) by fermentation. A higher concentration of SCFA was observed in Lf-treated piglets, and the group with higher SCFA also showed higher expression of *OCLN*. Based on results obtained by Olivier et al. [37], it was concluded that the tight junction may be upregulated as a consequence of higher levels of SCFAs, which activate the AMPK (5′ AMP-activated protein kinase) signaling pathway. Our study found no differences in the gene expression of *OCLN* in the small intestine; however, in the large intestine, we noticed a higher level of *OCLN* after 1 month (Col-t1) and lower after 3 months (Col-t3) of supplementation when compared with the control animals. Considering that most bacteria live in the large intestine, increased *OCLN* in the large intestine can be connected with the production of SCFA. However, the decreased level of *OCLN* expression at the second time point is surprising and perhaps related to age. 

#### 4.1.6. Cytoskeleton Components

Several studies indicate that Lf can induce the expression of the myosin heavy chain and promote myotube formation in myoblasts [38]. Since the cytoskeleton is a key element in assembling intercellular junctions, we considered it necessary to evaluate the expression level of *MYH9* and *ACTG2* in response to Col treatment. However, our data from the small intestine showed no difference in *MYH9* and a lack of differences in both the large and small intestine in *ACTG2* expression. Recently, human zonulin, a protein associated with the tight junction complex opening at the intestinal epithelium, was identified as prehaptoglobin-2 (pre-*HP*2) [39]. *HP* is a hemoglobin-binding protein with immunomodulatory properties; thus, we decided to check its expression after Col stimulation but found no statistical differences in its level at any analyzed time point.

### 4.2. Relationships in the Expression of Cell Adhesion Molecules

The obtained results allowed us to identify some interesting relationships in the expression of particular cell adhesion molecules. A variety of cell–matrix or cell–cell adhesion proteins are associated with the actin cytoskeleton. However, for many years, there was no evidence of a connection between connexins—fracture joints (that are both channels and adhesion molecules) and actin [40]. Later it was concluded that connexins may associate with actin to stabilize gap junctions at the plasma membrane. Our results in the small intestine found the strongest positive correlation between *CX43* and *ACTG2*, as well as *CX43* and *MYH9*. We did not find a similar relationship in the large intestine. Growing data indicate that Cx43 appears to act as a signaling step involved in actin polymerization and can have a critical role in the proper formation and alignment of actin-based contractile machinery [41,42]. It was shown that myosin aids in actin-mediated Cx43 accretion and that myosin VI is dispensable for the delivery of Cx43 to the cell surface and connexon movement in the plasma membrane in the heart tissue [43]. In our study, we found a strong positive correlation between *HP* and *CX43* levels in both the large and small intestines; however, this association seems to be stronger in the small intestine. It was demonstrated that the expression of *CX43* is critical for the normal hematopoiesis [44] and that *Hp* binds hemoglobin. We also found a strong positive correlation between *HP* and *ACTG2*, but only in the small intestine. This is an interesting observation as some studies suggest that Hp can induce changes in the cell morphology, actin cytoskeleton structure, and migration ability of tumor cells, thus supporting the induction of metastatic phenotype in these cells [45]. However, such a correlation in healthy cells may also be present to control the activity of the cellular cytoskeleton. In both the large and small intestines, we observed strong or moderate, respectively, interactions between *CDH2* and *CX43*. This is not surprising as *CX43* can act as a regulator of *CDH1* transcription [28]. Interestingly, in our study, we did not observe the age-dependent loss of Ctnnb or Cdh2 expressions that were found by Nichols et al. [46]. This could be explained by the short duration of our experiment. The correlation between *ACT2G* and *MYH9* that we observed should not be surprising, since the interactions between these proteins are the basis of the mechanism of contraction. A moderate correlation was observed between *VCL* and *ACTG2*, but once again it was stronger in the small intestine compared to in the large one. It was found that Vcl can interact with Actg in the recruitment of actin filaments to the growing focal adhesions, as well as in the capping of actin filaments to regulate actin dynamics [32]. Adhesive interactions of cadherins induce the crosstalk between the adhesion complexes and the actin cytoskeleton, allowing the strengthening of adhesions and cytoskeletal organization. These correlations were observed in both the large and small intestine; however, these were stronger in the large intestine.

### 4.3. The Limitation of the Present Study 

We are aware that the current study has several limitations. First, Col and its major component Lf is an unspecific factor with a broad spectrum of activity, and cell adhesion molecules are involved in many interactions; thus, it should be confirmed that the biological effects are truly caused by Col/Lf. Secondly, due to the preliminary character of the study, small study groups were used. Further studies with intestinal cell lines should be implemented as cell adhesion molecules change the intracellular pathway. We do not know the effects of Col supplementation in females because only males were used in the experiment. This was due to the fact that the female endocrine system is much more complex and would constitute an additional variable making it difficult to identify underlying observations. In our study, we did not observe statistically significant changes in the expression level of any of the intercellular junction proteins in the small or large intestine after 6 months of Col supplementation. This may mean that in the research model we used, the dose of Col used (100 mg/kg·bw/day, administered orally) for 1, 3, or 6 months was not sufficient to induce permanent changes in vivo or that Col does not induce such changes permanently in vivo. Another limitation of the study is a lack of parameters for the quantifying tight junction function; however, we focused on changes in gene and protein expression. The consequences and mechanisms of these changes can be included in a future study.

## 5. Conclusions

The present data indicate that Col can temporarily alter some components of the cell adhesion molecules involved in the formation of the cellular barrier. What surprised us most was the lack of statistically significant differences after the longest period of Col supplementation. In the future, it would be worth investigating why supplementation-induced changes in intercellular connections are not always stable and long-lasting, which may be important for many patients. Perhaps the use of Col supplements should only be considered for some periods, e.g., of increased activity, or of decreased immunity. In our work, we focused on observing changes, not their mechanisms. We hope that the molecular mechanism responsible for Col-mediated changes of protein regulating the epithelial barrier function and cross talk between the CJ components in the intestine in vivo will be implemented in our future studies. 

## Figures and Tables

**Table 1:** Results of a simple correlation between *CDH1*, *CDH2*, *CTNNB*1, *CX43*, *VCL*, *OCLN*, *HP*, and *ACTG2*, and the epithelium and microvilli thickness in the small intestine.

	*CDH1*	*CDH2*	*CTNNB1*	*ACTG2*	*VCL*	*CX43*	*HP*	*OCLN*	*MYH9*	Epithelium	Microvilli
*CDH1*		***r = 0.07*** ***p = 0.71***	***r = 0.61*** p < 0.01 *	***r = 0.04*** ***p = 0.85***	***r = 0.15*** ***p = 0.48***	***r = 0.03*** ***p = 0.87***	***r = −0.08*** ***p = 0.70***	***r = 0.36*** ***p = 0.08***	***r = 0.48*** p < 0.05 *	***r = −0.11*** ***p = 0.57***	***r = −0.12*** ***p = 0.57***
*CDH2*	***r = 0.07*** ***p = 0.71***		***r = 0.28*** ***p = 0.18***	***r = 0.74*** p < 0.01 *	***r = 0.62*** p < 0.01*	***r = 0.76*** p < 0.01 *	***r = 0.65*** p < 0.01 *	***r = 0.11*** ***p = 0.64***	***r = 0.61*** p < 0.01 *	***r = −0.25*** ***p = 0.22***	***r = 0.13*** ***p = 0.52***
*CTNNB1*	***r = 0.61*** p < 0.01 *	***r = 0.28*** ***p = 0.18***		***r = 0.52*** p < 0.01 *	***r = 0.56*** p < 0.01 *	***r = 0.42*** p < 0.05 *	***r = 0.46*** p < 0.05 *	***r = 0.44*** p < 0.05 *	***r = 0.72*** p < 0.01 *	***r = −0.10*** ***p = 0.63***	***r = −0.25*** ***p = 0.22***
*ACTG2*	***r = 0.04*** ***p = 0.85***	***r = 0.74*** p < 0.01 *	***r = 0.52*** p < 0.01 *		***r = 0.74*** p < 0.01 *	***r = 0.91*** p < 0.01 *	***r = 0.81*** p < 0.01 *	***r = 0.35*** ***p = 0.09***	***r = 0.76*** p < 0.01 *	***r = −0.35*** ***P = 0.09***	***r = 0.12*** ***p = 0.56***
*VCL*	***r = 0.15*** ***p = 0.48***	***r = 0.62*** p < 0.01 *	***r = 0.56*** p < 0.01 *	***r = 0.74*** p < 0.01 *		***r = 0.61*** p < 0.01 *	***r = 0.61*** p < 0.01 *	***r = 0.18*** ***p = 0.38***	***r = 0.46*** p < 0.05 *	***r = 0.03*** ***p = 0.89***	***r = −0.33*** ***p = 0.10***
*CX43*	***r = 0.03*** ***p = 0.87***	***r = 0.76*** p < 0.01 *	***r = 0.42*** p < 0.05 *	***r = 0.91*** p < 0.01 *	***r = 0.61*** p < 0.01 *		***r = 0.83*** ***p < 0.01***	***r = 0.34*** ***p = 0.10***	***r = 0.77*** p < 0.01 *	***r = −0.40*** p < 0.05 *	***r = 0.27*** ***p = 0.20***
*HP*	***r = −0.08*** ***p = 0.70***	***r = 0.65*** p < 0.01 *	***r = 0.46*** p < 0.05 *	***r = 0.81*** p < 0.01 *	***r = 0.61*** p < 0.01 *	***r = 0.83*** ***p < 0.01***		***r = 0.23*** ***p = 0.28***	***r = 0.66*** ***p < 0.01***	***r = −0.19*** ***p = 0.38***	***r = 0.14*** ***p = 0.51***
*OCLN*	***r = 0.36*** ***p = 0.08***	***r = 0.11*** ***p = 0.64***	***r = 0.44*** p < 0.05 *	***r = 0.35*** ***p = 0.09***	***r = 0.18*** ***p = 0.38***	***r = 0.34*** ***p = 0.10***	***r = 0.23*** ***p = 0.28***		*r = 0.53* p < 0.01 *	*r = −0.31* *p = 0.12*	***r = 0.21*** ***p = 0.33***
*MYH9*	***r = 0.48*** p < 0.05 *	***r = 0.61*** p < 0.01 *	***r = 0.72*** p < 0.01 *	***r = 0.76*** p < 0.01 *	***r = 0.46*** p < 0.05 *	***r = 0.77*** p < 0.01 *	***r = 0.66*** p < 0.01 *	*r = 0.53* p < 0.01 *		*r = −0.39* p < 0.05 *	***r = 0.20*** ***p = 0.33***
Epithelium	***r = −0.11*** ***p = 0.57***	***r = −0.25*** ***p = 0.22***	***r = −0.10*** ***p = 0.63***	***r = −0.35*** ***P = 0.09***	***r = 0.03*** ***p = 0.89***	***r = −0.40*** p < 0.05 *	***r = −0.19*** ***p = 0.38***	*r = −0.31* *p = 0.12*	*r = −0.39* p < 0.05 *		***r = −0.32*** ***p = 0.11***
Microvilli	***r = −0.12*** ***p = 0.57***	***r = 0.13*** ***p = 0.52***	***r = −0.25*** ***p = 0.22***	***r = 0.12*** ***p = 0.56***	***r = −0.33*** ***p = 0.10***	***r = 0.27*** ***p = 0.20***	***r = 0.14*** ***p = 0.51***	***r = 0.21*** ***p = 0.33***	***r = 0.20*** ***p = 0.33***	***r = −0.32*** ***p = 0.11***	

Spearman correlation coefficient (for data that did not pass the normality test, bold font); Pearson correlation coefficient (for data that passed normality test, normal font). * *p* (two-tailed) ≤ 0.05. Correlation coefficient ≥ 0.7 is marked with red.

**Table 2:** Results of a simple correlation between *CDH1*, *CDH2*, *CTNNB1*, *CX43*, *VCL*, *OCLN*, *HP*, *ACTG2*, the epithelium, and microvilli in the large intestine.

	*CDH1*	*CDH2*	*CTNNB1*	*ACTG2*	*VCL*	*CX43*	*HP*	*OCLN*	Epithelium	Microvilli
*CDH1*		*r = 0.55* *p < 0.01* *	***r = 0.49*** ***p = 0.01*** *	*r = 0.24* *p = 0.22*	*r = 0.26* *p =0.18*	*r = 0.25* *p = 0.20*	***r = −0.13*** ***p = 0.51***	***r = 0.67*** ***p < 0.01*** *	*r = 0.42* *p = 0.02* *	***r = 0.28*** ***p = 0.15***
*CDH2*	*r = 0.55* *p < 0.01* *		***r = 0.38*** ***p = 0.06***	*r = 0.56* *p < 0.01* *	*r = 0.75* *p < 0.01* *	*r = 0.49* *p < 0.01* *	***r = 0.19*** ***p = 0.33***	***r = 0*** ***p > 0.99***	*r = 0.47* *p = 0.01* *	***r = 0.13*** ***p = 0.56***
*CTNNB1*	***r = 0.49*** ***p = 0.01*** *	***r = 0.38*** ***p = 0.06***		***r = 0.28*** ***p = 0.15***	***r = 0.45*** ***p = 0.02***	***r = 0.36*** ***p = 0.06***	***r = 0.22*** ***p = 0.27***	***r = 0.08*** ***p = 0.68***	***r = 0.03*** ***p = 0.85***	***r = 0.03*** ***p = 0.86***
*ACTG2*	*r = 0.24* *p = 0.22*	*r = 0.56* *p < 0.01* *	***r = 0.28*** ***p = 0.15***		*r = 0.62* *p < 0.01* *	*r = 0.21* *p = 0.31*	***r = 0.04*** ***p = 0.81***	***r = −0.27*** ***p = 0.16***	*r = 0.06* *p = 0.74*	***r = 0.03*** ***p = 0.86***
*VCL*	*r = 0.26* *p =0.18*	*r = 0.75* *p < 0.01* *	***r = 0.45*** ***p = 0.02*** *	*r = 0.62* *p < 0.01* *		*r = 0.30* *p =0.12*	***r = 0.15*** ***p = 0.42***	***r = −0.20*** ***p = 0.31***	*r = 0.27* *p = 0.16*	***r = 0.13*** ***p = 0.52***
*CX43*	*r = 0.25* *p = 0.20*	*r = 0.49* *p < 0.01* *	***r = 0.36*** ***p = 0.06***	*r = 0.21* *p = 0.31*	*r = 0.30* *p =0.12*		***r = 0.71*** ***p < 0.01*** *	***r = −0.22*** ***p = 0.27***	*r = 0.15* *p = 0.42*	***r = 0.19*** ***p = 0.33***
*HP*	***r = −0.13*** ***p = 0.51***	***r = 0.19*** ***p = 0.33***	***r = 0.22*** ***p = 0.27***	***r = 0.04*** ***p = 0.81***	***r = 0.15*** ***p = 0.42***	***r = 0.71*** ***p < 0.01*** *		***r = −0.35*** ***p = 0.06***	***r = −0.29*** ***p = 0.13***	***r < 0.01*** ***p = 0.99***
*OCLN*	***r = 0.67*** ***p < 0.01*** *	***r = 0*** ***p > 0.99***	***r = 0.08*** ***p = 0.68***	***r = −0.27*** ***p = 0.16***	***r = −0.20*** ***p = 0.31***	***r = −0.22*** ***p = 0.27***	***r =*** ***−*** ***0.35*** ***p = 0.06***		***r = 0.41*** ***p = 0.03*** *	***r = 0.44*** ***p = 0.02*** *
Epithelium	*r = 0.42* *p = 0.02* *	*r = 0.47* *p = 0.01* *	***r = 0.03*** ***p = 0.85***	*r = 0.06* *p = 0.74*	*r = 0.27* *p = 0.16*	*r = 0.15* *p = 0.42*	***r = −0.29*** ***p = 0.13***	***r = 0.41*** ***p = 0.03*** *		***r = 0.33*** ***p = 0.08***
Microvilli	***r = 0.28*** ***p = 0.15***	***r = 0.13*** ***p = 0.56***	***r = 0.03*** ***p = 0.86***	***r < 0.01*** ***p = 0.99***	***r = 0.13*** ***p = 0.52***	***r = 0.19*** ***p = 0.33***	***r < 0.01*** ***p = 0.99***	***r = 0.44*** ***p = 0.02*** *	***r = 0.33*** ***p = 0.08***	

Spearman correlation coefficient (for data that did not pass the normality test, bold font); Pearson correlation coefficient (for data that passed normality test, normal font). * *p* (two-tailed) ≤ 0.05. Correlation coefficient ≥ 0.7 is marked with red.

## Data Availability

The data presented in this study are available on request from the corresponding author.

## Supplementary Materials

The following supporting information can be downloaded at: https://www.mdpi.com/article/10.3390/foods11050685/s1. Figure S1: The charts show the dCt, the fold change of gene expression (relative to housekeeping gene GAPDH) in the small intestine which were not significant. Figure S2: The charts show the dCt, the fold change of gene expression (relative to housekeeping gene GAPDH) in the large intestine which were not significant.

## References

[1] Lee B., Moon K.M., Kim C.Y. (2018). Tight junction in the intestinal epithelium: Its association with diseases and regulation by phytochemicals. J. Immunol. Res..

[2] Suzuki T. (2020). Regulation of the intestinal barrier by nutrients: The role of tight junctions. Anim. Sci. J..

[3] Lepanto M.S., Rosa L., Paesano R., Valenti P., Cutone A. (2019). Lactoferrin in aseptic and septic inflammation. Molecules.

[4] Santos-Pereira C., Rocha J.F., Fernandes H.S., Rodrigues L.R., Côrte-Real M., Sousa S.F. (2021). The milk-derived lactoferrin inhibits V-ATPase activity by targeting its V1 domain. Int. J. Biol. Macromol..

[5] Alexander M.R., Norlander A., Elijovich F., Atreya R.V., Gaye A., Gnecco J.S., Laffer C.L., Galindo C.L., Madhur M.S. (2019). Human monocyte transcriptional profiling identifies IL-18 receptor accessory protein and lactoferrin as novel immune targets in hypertension. Br. J. Pharmacol..

[6] Bagwe-Parab S., Yadav P., Kaur G., Tuli H.S., Buttar H.S. (2020). Therapeutic applications of human and bovine colostrum in the treatment of gastrointestinal diseases and distinctive cancer types: The current evidence. Front. Pharmacol..

[7] Huang L., Chen R., Liu L., Zhou Y., Chen Z. (2021). Lactoferrin ameliorates pathological cardiac hypertrophy related to mitochondrial quality control in aged mice. Food Funct..

[8] Trybek G., Metlerski M., Szumilas K., Aniko-Włodarczyk M., Preuss O., Grocholewicz K., Wiszniewska B. (2016). The biological properties of lactoferrin. Cent. Eur. J. Sport Sci. Med..

[9] Trybek G., Jedliński M., Jaroń A., Preuss O., Mazur M., Grzywacz A. (2020). Impact of lactoferrin on bone regenerative processes and its possible implementation in oral surgery—A systematic review of novel studies with metanalysis and metaregression. BMC Oral Health.

[10] Cutone A., Rosa L., Ianiro G., Lepanto M.S., Di Patti M.C.B., Valenti P., Musci G. (2020). Lactoferrin’s anti-cancer properties: Safety, selectivity, and wide range of action. Biomolecules.

[11] Zhao X., Xu X.-X., Liu Y., Xi E.-Z., An J.-J., Tabys D., Liu N. (2019). The in vitro protective role of bovine lactoferrin on intestinal epithelial barrier. Molecules.

[12] Kur P., Kolasa-Wołosiuk A., Grabowska M., Kram A., Tarnowski M., Baranowska-Bosiacka I., Rzeszotek S., Piasecka M., Wiszniewska B. (2021). The postnatal offspring of finasteride-treated male rats shows hyperglycaemia, elevated hepatic glycogen storage and altered GLUT2, IR, and AR expression in the liver. Int. J. Mol. Sci..

[13] Liu H.-M., Yang D., Liu Z.-F., Hu S.-Z., Yan S.-H., He X.-W. (2019). Density distribution of gene expression profiles and evaluation of using maximal information coefficient to identify differentially expressed genes. PLoS ONE.

[14] Schneider M.R., Dahlhoff M., Horst D., Hirschi B., Trülzsch K., Müller-Höcker J., Vogelmann R., Allgäuer M., Gerhard M., Steininger S. (2010). A key role for e-cadherin in intestinal homeostasis and paneth cell maturation. PLoS ONE.

[15] Nelson W.J., Nusse R. (2004). Convergence of Wnt, beta-catenin, and cadherin pathways. Science.

[16] Zadvornyi T.V., Lukianova N.Y., Borikun T.V., Chekhun V.F. (2018). Effects of exogenous lactoferrin on phenotypic profile and inva-siveness of human prostate cancer cells (DU145 and LNCaP) in vitro. Exp. Oncol..

[17] Kline K.T., Lian H., Zhong X.S., Luo X., Winston J.H., Cong Y., Savidge T.C., Dashwood R.H., Powell D.W., Li Q. (2020). Neonatal injury increases gut permeability by epigenetically suppressing E-cadherin in adulthood. J. Immunol..

[18] Cepek K.L., Shaw S.K., Parker C.M., Russell G.J., Morrow J.S., Rimm D.L., Brenner M.B. (1994). Adhesion between epithelial cells and T lymphocytes mediated by E-cadherin and the αEβ7 integrin. Nature.

[19] Grabowska M.M., Day M.L. (2012). Soluble E-cadherin: More than a symptom of disease. Front. Biosci..

[20] Trentini A., Maritati M., Cervellati C., Manfrinato M.C., Gonelli A., Volta C.A., Vesce F., Greco P., Dallocchio F., Bellini T. (2016). Vaginal lactoferrin modulates PGE2, MMP-9, MMP-2, and TIMP-1 amniotic fluid concentrations. Mediat. Inflamm..

[21] Libusova L., Stemmler M.P., Hierholzer A., Schwarz H., Kemler R. (2010). N-cadherin can structurally substitute for E-cadherin during intestinal development but leads to polyp formation. Development.

[22] Loh C.-Y., Chai J.Y., Tang T.F., Wong W.F., Sethi G., Shanmugam M.K., Chong P.P., Looi C.Y. (2019). The E-cadherin and N-cadherin switch in epithelial-to-mesenchymal transition: Signaling, therapeutic implications, and challenges. Cells.

[23] Takayama Y., Mizumachi K. (2010). Inhibitory effect of lactoferrin on hypertrophic differentiation of ATDC5 mouse chondroprogenitor cells. BioMetals.

[24] Liu J., Li B., Lee C., Zhu H., Zheng S., Pierro A. (2019). Protective effects of lactoferrin on injured intestinal epithelial cells. J. Pediatr. Surg..

[25] Zhang M., Chen C.Y., Hu Y., Lyu B. (2020). The relationship between adherens junction and tight junction and clinical symptoms in patients with diarrhea predominant irritable bowel syndrome. Zhonghua Nei Ke Za Zhi.

[26] Maes M., Yanguas S.C., Willebrords J., Cogliati B., Vinken M. (2015). Connexin and pannexin signaling in gastrointestinal and liver disease. Transl. Res..

[27] Al-Ghadban S., Kaissi S., Homaidan F.R., Naim H.Y., El-Sabban M.E. (2016). Cross-talk between intestinal epithelial cells and immune cells in inflammatory bowel disease. Sci. Rep..

[28] Kotini M., Barriga E.H., Leslie J., Gentzel M., Rauschenberger V., Schambony A., Mayor R. (2018). Gap junction protein connexin-43 is a direct transcriptional regulator of N-cadherin in vivo. Nat. Commun..

[29] Yang S.-C., Hung C.-F., Aljuffali I.A., Fang J.-Y. (2015). The roles of the virulence factor IpaB in Shigella spp. in the escape from immune cells and invasion of epithelial cells. Microbiol. Res..

[30] Vandrovcova M., Douglas T.E.L., Heinemann S., Scharnweber D., Dubruel P., Bacakova L. (2015). Collagen-lactoferrin fibrillar coatings enhance osteoblast proliferation and differentiation. J. Biomed. Mater. Res. Part A.

[31] Adler B.L., McMahan Z. (2021). Anti-vinculin autoantibodies in systemic sclerosis: A step toward a novel biomarker?. Clin. Rheumatol..

[32] Pimentel M., Morales W., Pokkunuri V., Brikos C., Kim S.M., Kim S.E., Triantafyllou K., Weitsman S., Marsh Z., Marsh E. (2014). Autoimmunity links vinculin to the pathophysiology of chronic functional bowel changes following campylobacter jejuni infection in a rat model. Am. J. Dig. Dis..

[33] Mierke C.T., Kollmannsberger P., Zitterbart D.P., Diez G., Koch T.M., Marg S., Ziegler W.H., Goldmann W.H., Fabry B. (2010). Vinculin Facilitates Cell Invasion into Three-dimensional Collagen Matrices. J. Biol. Chem..

[34] Zhang C.H., Geng J.S. (2007). Expression of paxillin and vinculin in gastric carcinoma and precancerous lesion and their effects on prognosis of gastric carcinoma. Chin. J. Diagn. Pathol..

[35] Oh-Oka K., Kono H., Ishimaru K., Miyake K., Kubota T., Ogawa H., Okumura K., Shibata S., Nakao A. (2014). Expressions of tight junction proteins occludin and claudin-1 are under the circadian control in the mouse large intestine: Implications in intestinal permeability and susceptibility to colitis. PLoS ONE.

[36] Hu P., Zhao F., Wang J., Zhu W. (2020). Early-life lactoferrin intervention modulates the colonic microbiota, colonic microbial metabolites and intestinal function in suckling piglets. Appl. Microbiol. Biotechnol..

[37] Olivier S., Leclerc J., Grenier A., Foretz M., Tamburini J., Viollet B. (2019). AMPK activation promotes tight junction assembly in intestinal epithelial caco-2 cells. Int. J. Mol. Sci..

[38] Kitakaze T., Oshimo M., Kobayashi Y., Ryu M., Suzuki Y.A., Inui H., Harada N., Yamaji R. (2018). Lactoferrin promotes murine C2C12 myoblast proliferation and differentiation and myotube hypertrophy. Mol. Med. Rep..

[39] Vanuytsel T., Vermeire S., Cleynen I. (2013). The role of haptoglobin and its related protein, zonulin, in inflammatory bowel disease. Tissue Barriers.

[40] Wall M.E., Otey C., Qi J., Banes A.J. (2007). Connexin 43 is localized with actin in tenocytes. Cell Motil. Cytoskelet..

[41] Ionta M., Ferreira R.A.S., Pfister S.C., Machado-Santelli G.M. (2009). Exogenous Cx43 expression decrease cell proliferation rate in rat hepatocarcinoma cells independently of functional gap junction. Cancer Cell Int..

[42] Batra N., Burra S., Siller-Jackson A.J., Gu S., Xia X., Weber G.F., DeSimone D., Bonewald L.F., Lafer E.M., Sprague E. (2012). Mechanical stress-activated integrin 5 1 induces opening of connexin 43 hemichannels. Proc. Natl. Acad. Sci. USA.

[43] Waxse B.J., Sengupta P., Hesketh G.G., Lippincott-Schwartz J., Buss F. (2017). Myosin VI facilitates connexin 43 gap junction accretion. J. Cell Sci..

[44] Montecino-Rodriguez E., Leathers H., Dorshkind K. (2000). Expression of connexin 43 (Cx43) is critical for normal hematopoiesis. Blood.

[45] Garibay-Cerdenares O.L., I Hernández-Ramírez V., Osorio-Trujillo J.C., Gallardo-Rincón D., Talamás-Rohana P. (2015). Haptoglobin and CCR2 receptor expression in ovarian cancer cells that were exposed to ascitic fluid: Exploring a new role of haptoglobin in the tumoral microenvironment. Cell Adhes. Migr..

[46] Nichols L.A., Grunz-Borgmann E.A., Wang X., Parrish A.R. (2014). A role for the age-dependent loss of α(E)-catenin in regulation of N-cadherin expression and cell migration. Physiol. Rep..

