# Supplementary material for: Colostrum-Induced Temporary Changes in the Expression of Proteins Regulating the Epithelial Barrier Function in the Intestine
Source: Foods. 2022 Feb 25;11(5):685. doi: 10.3390/foods11050685 (PMC8909690; doi:10.3390/foods11050685)
Supplement: Supplementary file 1 [file foods-11-00685-s001.zip › foods-1581725-supplementary.pdf]

# Supplementary Materials

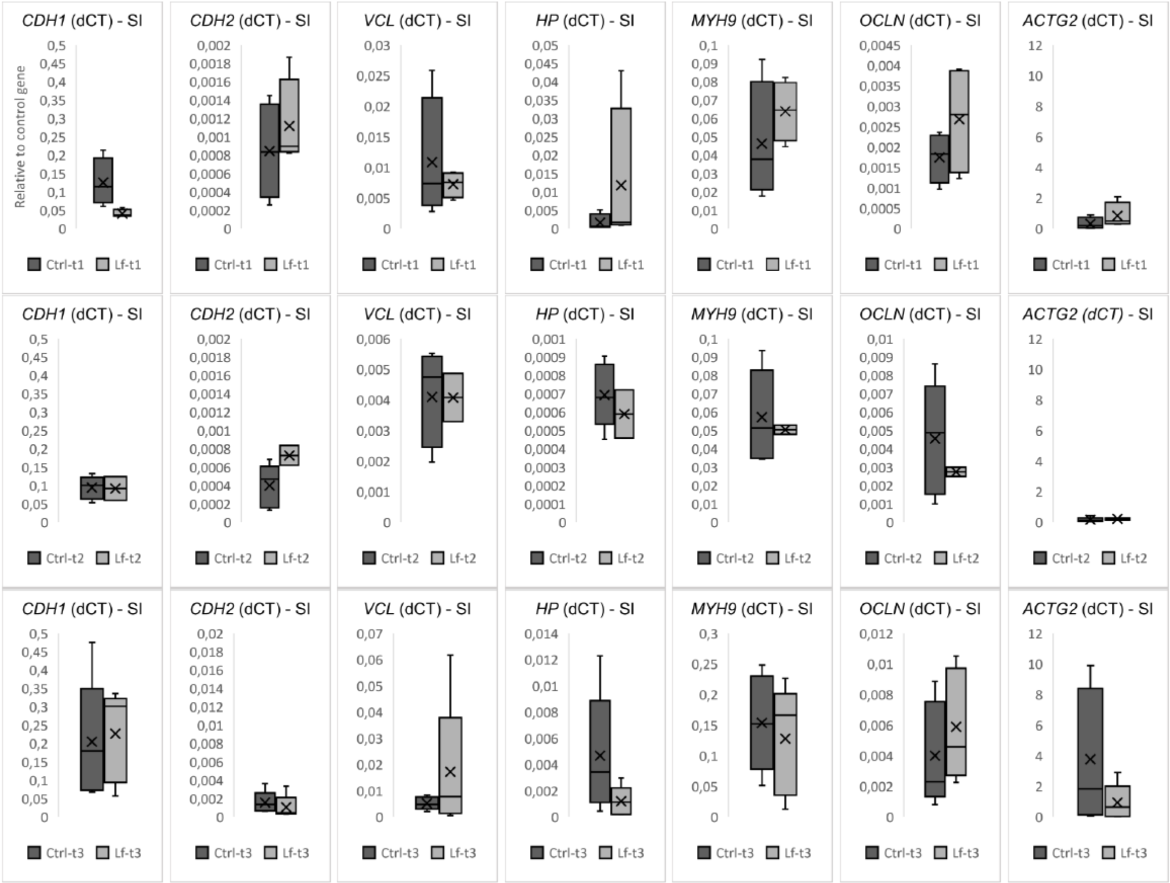


**Figure S1.** The charts show the dCt, the fold change of gene expression (relative to housekeeping gene GAPDH) in the small intestine which were not significant.

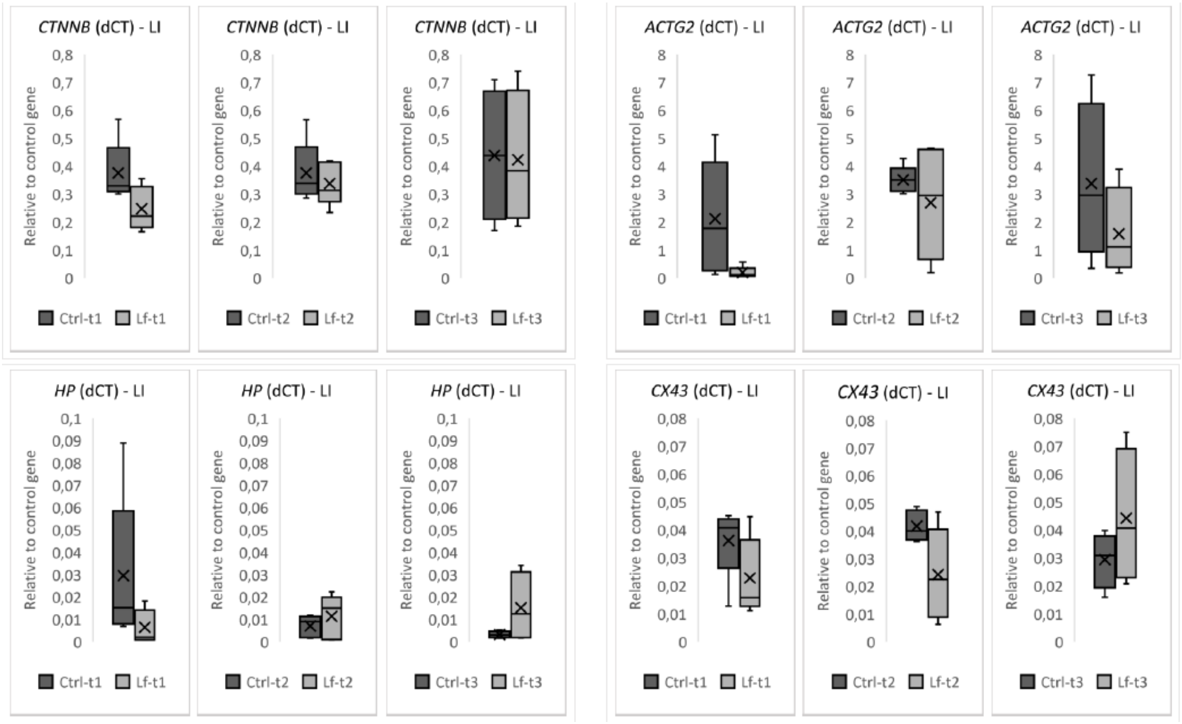


**Figure S2.** The charts show the dCt, the fold change of gene expression (relative to housekeeping gene GAPDH) in the large intestine which were not significant.
